# Can Weight Watchers (WW) Help Address Maternal Obesity? An Audit of Weight Change in Women of Childbearing Age and Mothers-To-Be, Referred into a Commercial Slimming Programme

**DOI:** 10.1007/s10995-021-03204-5

**Published:** 2021-11-05

**Authors:** Karen Tocque, Lynne Kennedy

**Affiliations:** 1 Department of Clinical Sciences, University of Chester, Parkgate Road, Chester, CH1 4BJ UK; 2 KT Intelligence CIC, Wales, UK

**Keywords:** Obesity, BMI, Planned pregnancy, Weight-management, Intervention, Wales

## Abstract

**Objectives:**

The scale of overweight and obesity amongst women of childbearing age or mothers to be, living in Wales, places a considerable burden on the NHS and public health. High BMI (over 30) during pregnancy increases the health risks for mother and baby. Policy advice recommends weight management services are available to help women lose weight before and whilst planning pregnancy. In parts of Wales, NHS partnerships with commercial companies provide weight management services for women considering or planning pregnancy. This study evaluates whether an established referral Weight Watchers (WW) programme, known to be effective in adults in England, can help mothers-to-be living in North Wales lose weight.

**Methods:**

Analysis used routine data from 82 referrals to WW between June 2013 and January 2015. Participants received a referral letter inviting them to attend face-to-face group workshops combined with a digital experience. The programme encompassed healthy eating, physical activity and positive mind-set. Trained WW staff measured bodyweight before, during and at 12 weeks. On entry to the course, participants had a median age of 31.4 years (interquartile range (IQR) 28–34) with a median BMI of 36.8 kg/m^2^ (IQR 33.3–43.7).

**Results:**

Women completing the course (n = 34) had a median weight loss of 5.65 kg (IQR 0.45–10.85), equating to 5.7% (SD 3.46) of initial body weight. Intention-to-treat analysis (last observation carried forward), which included lapsed courses n = 66, showed a median weight loss of 3.6 kg (IQR − 2.53 to 9.73), equating to 3.7% (SD 3.62) of initial body weight. Overall, there was significant weight loss during the WW programme (Wilcoxon signed rank test Z = − 6.16; p < 0.001). Weight loss was significantly correlated with the number of workshops attended (Spearman correlation coefficient 0.61 p < 0.001). The proportion of all 82 participants (intention to treat, baseline observation carried forward) that achieved a weight loss of ≥ 5% initial weight was 30.5%.

**Conclusions for Practice:**

Referral of obese mothers-to-be into WW can successfully achieve short-term weight loss, at or above 5%, in approximately one third of participants. The dose–response effect supports a causal inference. Successful weight loss at this critical life stage may provide women with the necessary motivation to initiate weight loss for healthy pregnancy, however further research is required.

## Background

The foundation for good health starts in pregnancy; with the life chances of a child determined by the mother’s health, lifestyle and social circumstances (Marmot, 2010) and the intergenerational effect of maternal and childhood obesity having serious implications for the health of the mother, child and family (NICE, 2018). In the UK, the scale of maternal obesity (Body Mass Index ≥ 30 kg/m^2^) prior to and during pregnancy has become a major public health concern (CMACE, 2010) resulting in renewed attention on obesity prevention as part of maternity services (Denison et al., 2018).

Global trends for high-income countries suggest approximately one third of women of childbearing age are likely to be either overweight or obese (Chen et al., 2018) and in the UK, more than half of all women are classified as such (HSCIC, 2018). Women in Wales are more likely to be obese during pregnancy compared with the rest of the UK (CMACE, 2011). According to Maternity Services in North Wales (personal communication), in 2012 maternal obesity 27% of women classified as obese (BMI ≥ 30) at the start of their pregnancy; with 11.6% of these women with BMI ≥ 35. Thus, enhanced perinatal and maternity services including weight management support need to be developed for women of childbearing age and mothers-to-be (Denison et al., 2018).

Pre-pregnancy weight and body mass index (BMI) are important predictors of health related risks to mother, foetus and child, and leads to increased health service usage and healthcare costs during pregnancy (Morgan & Rahman et al., 2014). Obese pregnant women and their babies experience significantly greater risks compared with women of a healthy BMI (Boudet-Berquier et al., 2017), including gestational diabetes (Hilden et al., 2016; Meehan et al., 2014) pre-eclampsia, caesarean sections, anaesthetic complications, wound infections, stillbirth, congenital abnormalities and neonatal death (Boudet-Berquier et al., 2017). Obese mothers tend to experience complications during childbirth and are less likely to breastfeed (Wojcicki, 2011), whilst children of obese parents are more likely to become overweight in adulthood (Godfrey et al., 2017).

National guidance on the prevention or management of maternal obesity recommend behavioural interventions for women of child-bearing age, particularly women considering pregnancy in the near future, involving a broad multi-level approach (NICE, 2014a, 2014b). Moreover, pregnancy is a critical stage in the life course in terms of influencing positive lifestyle behaviours; arguably women who are pregnant or planning a pregnancy are more receptive to health messages (Smith et al., 2012). Thus it’s an ideal opportunity to effectively engage women of childbearing age and mothers-to-be in discussions about weight management.

However, some researchers (e.g. Furber & McGowan, 2011; Kocełak et al., 2012; Nyman et al., 2010) have identified heightened sensitivity to size amongst women who are obese during pregnancy and advise caution around implementing interventions, even interventions delivered by experienced and trusted health care professionals, including midwives. Client centred approaches are more effective when addressing sensitive lifestyle messages and there should be an emphasis on providing sensitive, non-judgemental communication (Powell & Hughes, 2012).

Amidst the mounting evidence of identifiable risks linked with maternal obesity, NHS services are required to implement measures to manage these risks and improve outcomes for mothers-to-be and obese pregnant women and their babies (Gaillard, 2015; Godfrey et al., 2017). Health care professionals in maternity services want to support women of childbearing age maintain a healthy weight but have limited time and training (Basu et al., 2014). Furthermore, maternity services in Wales are indemnified (NHS Welsh Risk Pool Services, 2011–2012), in relation to formal claims for clinical negligence or personal injury and assesses clinical standards specific to maternity services; evidence of approved written policy procedures, pathways or guidelines specific to the management of women with obesity, in pregnancy, are now required; stating that as part of their initial risk assessment, all mothers should be offered advice about diet, exercise and weight management, alongside discussion of the associated risks of a raised BMI in pregnancy (Basu et al., 2014). However, as research (e.g. Nyman et al., 2010) suggests, clinical consultations are time constrained; potential opportunities to raise or indeed counsel, on sensitive topics including weight, limited. The National Institute for Health and Care Excellence (NICE) NICE, 2014b, 2018), issued guidance to prompt improvements in the prevention and management of maternal obesity. This guidance responds to national concerns over increased mortality during pregnancy, including ‘Why Mothers Die’ (CEMACH, 2004), ‘*Saving Mothers Lives*’ (CEMACH, 2007), and ‘*Perinatal Mortality 2009*’ (CMACE, 2011). As a result, national guidance includes recommendations outlining the importance of measuring weight, height and calculating BMI at the initial antenatal assessment, discussing the risks associated with obesity in pregnancy, and enabling access to appropriate and accurate advice on nutrition, physical activity and weight management during pregnancy.

Additionally, Public Health Wales has introduced policies for women of child bearing age and mothers-to-be, recommending that weight loss in women with a BMI of 30 or above, should be encouraged, *prior to conception*. Moreover, these women should be offered interventions, underpinned by evidence-based behaviour change techniques (NICE, 2014b) as part of locally coordinated lifestyle and weight management services.

One such intervention is NHS referral to a WW programme. In England, this programme is known to lead to a weight loss of ≥ 5% in a third of adults and to be cost effective (Ahern et al, 2011). A pilot scheme, *‘Shape Up For The Future’*, was developed and implemented by Public Health in North Wales (Flintshire County), to deliver a weight management intervention to women of child bearing age or ‘mothers-to-be’ with a BMI ≥ 30, in partnership with and using the WW model. At the time of audit, NHS referral into WW was not widely adopted in Wales, so collection of Welsh-specific data was important to evaluate the effectiveness of the programme with this particular target. The aim of this study was to determine whether the local WW pilot resulted in successful weight loss in obese women of childbearing age*.*

## Methods

The pilot scheme was introduced in May 2013. All eligible participants referred into the scheme (see below) were offered places on the intervention at no cost to themselves. This scheme consisted of access to a 3-month programme from WW, 12 locally-based group WW meetings, combined with 16 weeks digital experience, supported by trained lay-people. Participants received free vouchers for 12 weekly supervised group sessions. At each meeting group members were weighed, there was a discussion led by the group leader and group members could share information and weight loss tips and set target weight loss. The programme encompassed healthy eating, physical activity and behaviour change components including creating a positive mind-set and relationship with food. Digital tools were provided through a mobile app and the WW website. At, the heart of Weight Watchers (WW) is its food point system; foods are assigned points depending on the calorific value and fat content, which increase point value, or fibre content, which decreases point value according to the individual’s daily point allowance. Additionally. physical activity is encouraged, members earn points with exercise for exchange against food points. This points system is designed to encourage moderate energy deficit, and weight-loss up to 2lbs/week. For details see Appendix 1. Data (see below) were collected by group leaders and relayed to a central database (weight was measured at workshops on calibrated scales). Participants were encouraged to aim for the goal of a weight loss of 5% of their initial body weight over the 12 sessions of the programme, and be motivated to maintain healthy lifestyle changes for themselves (and their families) during the project.

### Recruitment

The Flintshire Public Health team were responsible for screening women; recruiting them based upon referrals from local General Practitioners (GPs), fertility clinics and maternity services. The eligibility criteria included women resident in Flintshire, aged 18 + years, with a BMI ≥ 30, who were:

- Awaiting or receiving fertility treatment;
- Planning a pregnancy in the near future;
- Mothers of young children (aged < 5 years);
- Not currently planning a pregnancy, but who are motivated to be in ‘best shape’ for when they do want to conceive;
- Recent mothers who may be intending a further pregnancy.

*The exclusion criteria* Women were not eligible if they were < 18 years old; currently pregnant; had an eating disorder; or had been a paying member of WW in the 3 months.

### Data Collection

Trained WW leaders collected before and after anthropometric data (height, weight and derived BMI), details of patient demographics and attendance. Weight change was the primary outcome measure, with weight and BMI recorded at baseline and end visit.

A recruitment target of 90 women was established based on capacity in the 12 week programme. Initial screening involved talking to the clients about the scheme and what they could expect, and the recording of data on eligibility criteria around current or future plans for pregnancy, any fertility treatment or advice and numbers of existing children. Regular reviews of progress were made by submission of a dashboard to the Public Health team. This dashboard contained information on total referrals (broken down by participants who completed, active or lapsed) and summary information on BMI and weight loss.

### Data Analysis

An anonymised, password protected dataset was provided by WW, for all participants involved in the scheme. Postcode of residence was matched to Census geographic area,^1^ which was then allocated a social deprivation measure.^2^ This Wales Index of multiple deprivation (WIMD) is calculated from eight different domains (or types) of deprivation, each compiled from a range of different indicators. The demographics of the engaged participants were summarised with reference to what type of women engaged in respect to their family planning status. ‘Completers’ were defined in the audit as participants who attended 10 or more sessions of this 12 session intervention. Data were excluded where records were incomplete for height, weight, weight change or if there were discrepancies between course status and the number of workshops attended.

Intention-to-Treat [using BOCF (baseline observation carried forward) and LOCF (last observation carried forward)] was carried out to determine the median (and IQR) weight loss, % body weight change and BMI change. The number (and %) of completers, the frequency of 5% loss and associations with age, employment status, social deprivation and initial BMI were calculated.

The original screening questionnaire was used to assess motivation and family status in relation to weight loss success. In particular, whether planning for a future baby led to greater motivation for women to lose weight prior to conceiving was investigated. Analysis was undertaken using Microsoft Access and IBM SPSS Statistics v 23.

## Results

**Fig. 1:**
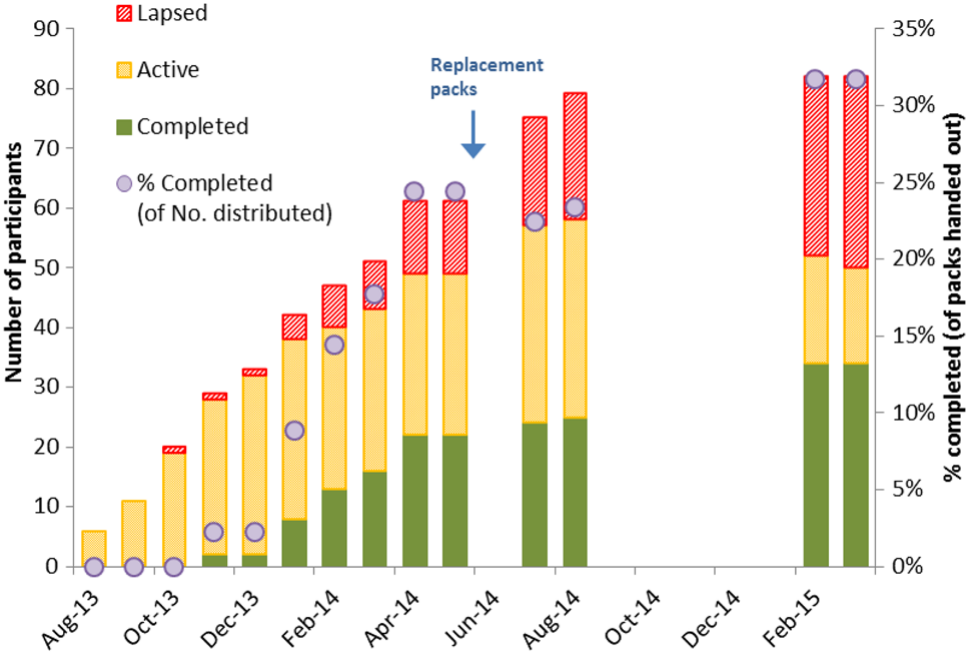
Correlation between weight change and attendance at workshops for the ‘Shape Up for the Future’ pilot

The first 90 referrals had been distributed by April 2014; at this time 22 (24%) of the participants had completed the course and 27 (30%) were still active (Fig. 1). WW reported that a proportion of the referrals (n = 25; 28%) had not been activated (i.e. potential participants did not attend). The data collection period was extended to January 2015 when a total of 82 / 107 (77%) referrals had been activated and 34 / 82 (41%) of courses had been completed; 32 (39%) had lapsed; and 16 (20%) were still active. Overall, 32% of the referrals had been completed.

Analysis of weight loss was undertaken on the 34 participants who successfully attended over 75% of the course (10–12 weeks). The 32 participants who had lapsed at some point during the course were included in intention-to-treat (ITT) analysis by applying the last observation carried forward (LOCF). Neither final nor latest weight measurements were available for the 16 courses still active but these individuals were included in ITT analysis using baseline observation carried forward (BOCF).

### Demographics of Participants

At the start of their courses, participants had a median age of 31.4 years (Interquartile range (IQR) 28–34) with a median BMI of 36.8 kg/m^2^ (IQR 33.3—43.7). Overall, 56% of women taking up the scheme were aged over 30 (Table 1). However, participants who completed the course were significantly more likely (79%) to be in this older group than participants who lapsed (44%) (Chi^2^ = 4.86, p < 0.05). In addition, women who lapsed were significantly more likely (53%) to be participants with the highest starting BMI compared with participants who completed the course (21%) (Chi^2^ = 7.6, p < 0.05). Active cases were not significantly different from completers or participants lapsed and none of the other demographics were significantly different between participant groups. Overall, 63% were employed, 96% were white, with an equal split across social deprivation areas (Table 1).

**Table 1:** Demographics of participants in the WW pilot

Group	Completed	%	Lapsed (ITT LOCF)	%	Active (ITT BOCF)	%	Total	%
*Age band*								
20–30	10	29	18	56	8	50	36	44
31–46	24	71	14	44	8	50	46	56
*Starting BMI (group)*								
27–33	10	29	5	16	5	31	20	24
34–39	17	50	10	31	10	63	37	45
40 +	7	21	17	53	1	6	25	30
*Employment status*								
Employed	22	65	21	66	9	56	52	63
Not given	6	18	5	16	4	25	15	18
Unemployed	6	18	6	19	3	19	15	18
*Ethnic category*								
White	34	100	30	94	15	94	79	96
Black African	0	0	0	0	1	6	1	1
Any other Black background	0	0	1	3	0	0	1	1
Info not obtained	0	0	1	3	0	0	1	1
*Social Deprivation (Wales IMD)*								
Least	11	32	6	19	5	31	22	27
2nd	8	24	6	19	5	31	19	23
3rd	4	12	9	28	0	0	13	16
Most	11	32	11	34	6	38	28	34
Total	34		32		16		82	
% of overall total	41		39		20			

### Weight Change During the Course

**Fig. 2:**
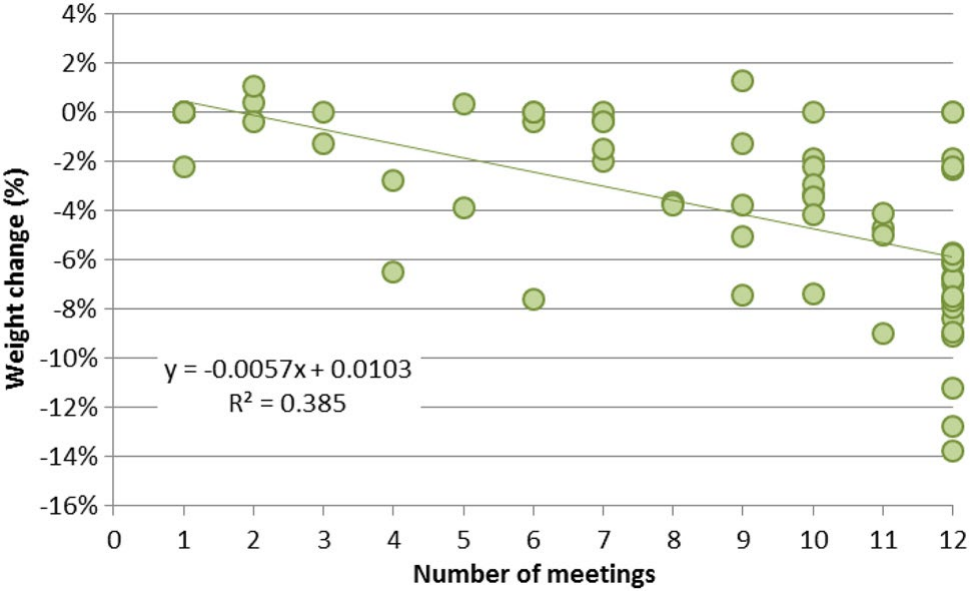
Timeline of recruitment and completion of the ‘Shape Up for the Future’ pilot

For completers and lapsed participants (ITT-LOCF), weight loss was significantly correlated with the number of workshops attended (Spearman correlation coefficient 0.61 p < 0.001; Fig. 2). R^2^ suggests that 39% of the variation in weight loss is associated with the number of workshops attended.

Correspondingly, the median weight and BMI loss increased with the number of workshops attended (Table 2) and were significantly higher in participants who attended all 12 workshops than in all other groups (Mann–Whitney U, p < 0.05).

**Table 2:** Average weight loss by number of workshops attended on the ‘Shape Up for the Future’ pilot

Status	Workshops attended	n	Weight loss (kg)		BMI decrease	
			Median	IQR	Median	IQR
Lapsed	1–2	10	0.00	0.17	0.00	0.08
3–6	10	0.90	4.95	0.95	2.23	
7–9	12	1.80	4.47	0.70	1.55	
Completed	10–11	12	3.60	2.75	1.30	0.98
12	22	6.80	4.40	2.40	1.60	
Overall	1–12	66	3.60	6.13	1.30	2.30
All completed	10–12	34	5.65	5.20	2.10	2.00

**Fig. 3:**
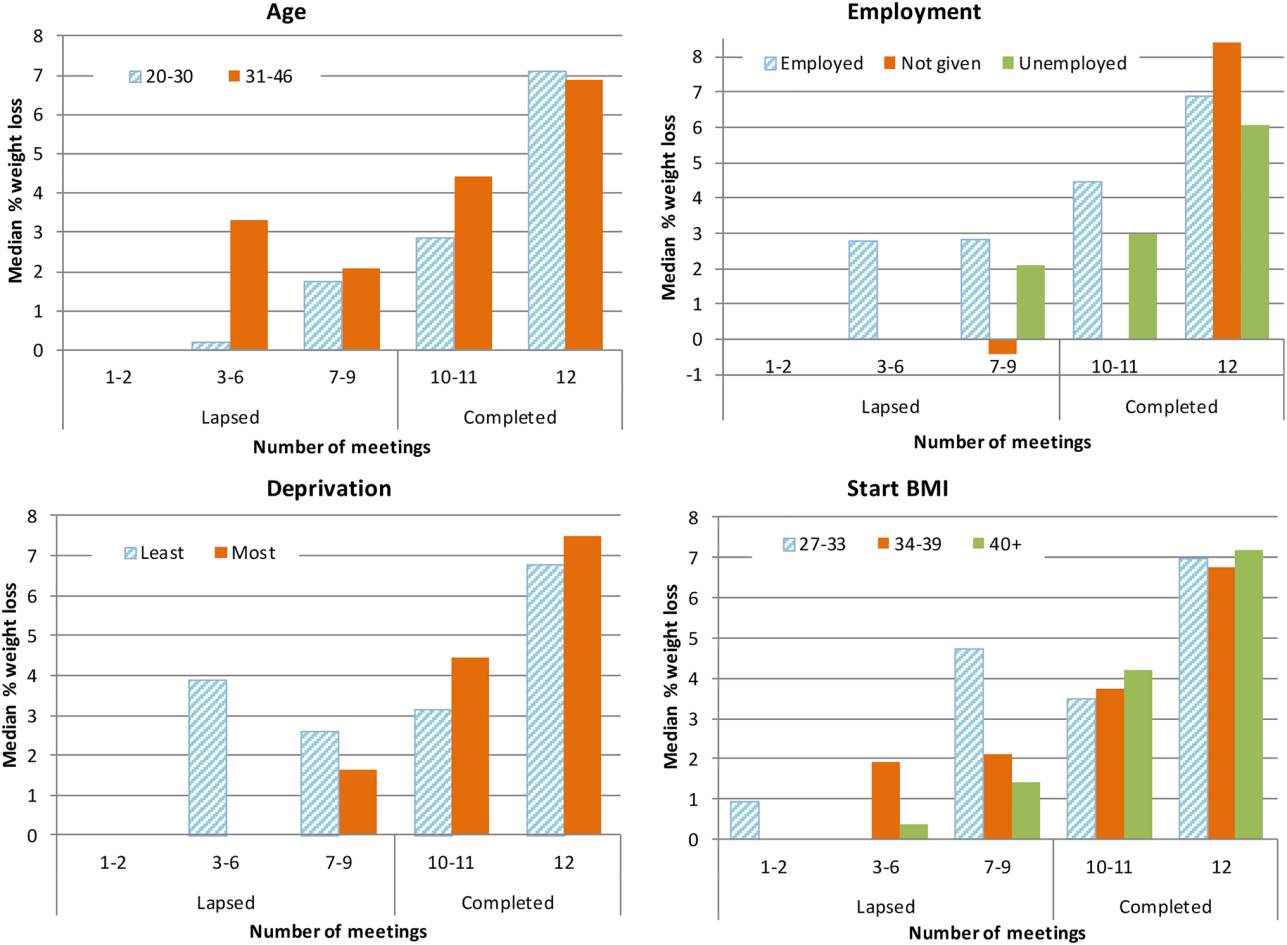
Average weight loss by various demographics of participants of the ‘Shape Up for the Future’ pilot

None of the other demographics (age, employment status, social deprivation) nor starting BMI showed any statistically significant difference in weight loss between completers and lapsed (Fig. 3). However, sample sizes were small when breaking down into sub-groups and so caution should be applied in interpreting these results. In addition, women stating a future intent to become pregnant in the screening questionnaire were no more likely to lose weight (64%) than participants not stating intent (80%).

### Successful Weight Loss

Of the total referrals to the programme (n = 82), i.e. on an intention-to-treat (baseline observation carried forward) basis, the proportion that achieved a weight loss of ≥ 5% initial weight was 30.5%, which included cases whose membership was still active and who had only initial weight recorded but no further weight recordings. This outcome achieves the recommended benchmarks for classifying an effective lifestyle weight management intervention (DOH, 2013).

For participants who had a weight change recorded at some point during the programme (completers or lapsed participants; n = 66), 38% achieved a weight loss of ≥ 5% initial weight, i.e. on an intention-to-treat (last observation carried forward) basis. Overall, these participants significantly lost weight during the WW programme (Wilcoxon signed rank test Z = -6.16; p < 0.001).

For completers only (n = 34), 59% achieved a weight loss of ≥ 5% initial weight. Participants who completed the course were significantly (Chi^2^p < 0.005) more likely to have lost over 5% initial weight than participants who lapsed (Table 3). Participants who lapsed were significantly (Chi^2^p < 0.05) more likely to have gained/maintained weight than participants completing the course.

**Table 3:** Weight loss success for various participants of the ‘Shape Up for the Future’ pilot

Weight Loss Category	Completers		Lapsed		Total (ITT LOCF)		Total (ITT BOCF)	
	n	%	n	%	n	%	n	%
Gained/maintained weight	3	9	13	41	16	24	16	20
Under 5% loss	11	32	14	44	25	38	25	30
5% and over loss	20	59	5	16	25	38	25	30
N =	34		32		66		82	

### Screening Data

Pregnancy intent (from screening data) was only available for 60% (n = 42) of participants and did not show any statistically significant relationship with successful weight loss. Moreover, women who stated a pregnancy intention were less likely to lose any weight (64%) than those not stating intent (81%) but also more likely to have a starting BMI of 40 + (38%) than those not stating pregnancy intention (23%). The small sample precludes any significance to be ascertained.

## Discussion

The NHS referral into WW in Flintshire was targeted at women of childbearing age with a BMI ≥ 30 kg/m^2^; including existing mothers who wanted to lose weight for health reasons or because they were considering pregnancy in the near future. This audit is therefore novel in investigating commercial weight loss programmes delivered in primary care to specifically support women of childbearing age to achieve or maintain a healthy weight.

The overall aim of the audit was to evaluate whether the programme achieved the recommended effectiveness of a lifestyle weight management intervention, where “At least 30% of all participants have achieved a weight loss equal to or greater than 5% of their initial weight at the end of the intervention” (DOH, 2013). Arguably, women at this stage in the life course, are considered to be receptive to messages and advice about healthy lifestyle including weight management. Intention for future pregnancy did not appear to impact on whether weight loss was achieved by participants in this programme. This is inconsistent with studies that suggest pregnancy is a critical life course factor in motivating women to prevent or reduce weight gain prior or during pregnancy respectively (CMACE, 2010; HSCIC, 2018; Smith et al., 2012). Our sample however, also included recent mothers whose motivations to lose weight do not relate to further or other pregnancy; diluting sample size. Given concerns about the lack of awareness or understanding in the general population over the importance of maintaining normal BMI in the preconception period this is an important contradiction. Further audit or research is recommended before accepting that women who are planning pregnancy and taking part in commercial weight loss programmes are any less motivated.

The initial target of 90 referrals was difficult to meet and after an extension of the scheme, 82 participants were recruited. The difficulty in recruitment is thought to be related to attempts at targeting overweight women who are attempting weight loss before embarking on another pregnancy. As research suggests (Nyman et al., 2010; Powell & Hughes, 2012), women in this group are less clear as to the nutritional advice for pre-pregnancy, gestational and postnatal periods. This finding concurs with appeals from professional bodies, such as the Royal College of Midwifery, for guidance on methods for healthy weight management in pregnancy (RCM, 2018).

Despite relatively small numbers, the audit was able to demonstrate success in initiating weight loss (i.e. overall 30% achieving ≥ 5% weight loss) by the Flintshire scheme and had similar retention and drop-out rates to the national evaluation of referral to WW (Ahern et al, 2011); almost half of the women (41%) completed the course (attending 10 or more sessions) and 39% lapsed at some point (1–9 sessions). In addition, over half (59%) of the women who did complete the course lost the target of 5% or more of their initial body weight; demonstrating that WW can lead to successful short-term weight loss, as defined by NICE guidance (NICE, 2018), whilst attending 10 or more workshops in the participation period. This success concurs with previous studies comparing the effectiveness of different commercial slimming programmes delivered in primary care (Heshka et al, 2003; Jolley et al, 2010). As an audit, this study has no control/comparison group and therefore further research would benefit from a case–control comparison in order to identify efficacy.

Without follow up data, it is not known whether these women maintained their lower weight or weight loss. Others (e.g. Ahern et al, 2011; Gudzune et al, 2015) have suggested WW attendees successfully maintain 2.9 kg of weight loss up to 2 years after attendance on the course. Since some women lapsed during the Flintshire course, actual success across all participants is lower; overall, almost a third (30.5%) of participants achieved a weight loss of ≥ 5% initial weight (ITT-BOCF), increasing to 39% of completers when combined with lapsed participants (ITT-LOCF). Comparable success has been reported by similar UK studies of community based commercial programmes (Jolley et al, 2010) and studies based in the US (Heshka et al, 2003). Some of these studies report recruitment strategies that may increase selection bias and therefore findings may not be typical of overweight women considering or planning pregnancy targeted by the present scheme.

Whilst other demographic factors might have influenced the success of weight loss in this study (age, social deprivation, ethnicity, BMI at baseline and employment status), sample sizes were too small to detect a significant impact. The findings are transferable to a white population (96% of participants, Caucasian British). Although ethnicity is consistent with the local context, as noted by Ahern et al (2016), uptake and engagement to similar commercial slimming programmes also experienced bias, attracting fewer males and socially deprived groups. The most consistent finding from this evaluation was that the number of workshops attended correlated significantly with weight loss. This type of dose–response effect is generally considered to support a causal relationship between the intervention and outcome (Reynolds, 1998) and is arguably (Wee et al., 2004) strongly correlated with structure and content (Moyer, 2012); according to a systematic review (Gudzune et al, 2015) and more recent studies of commercial open-group behavioural weight-management programmes, including WW, show evidence of effectiveness in RCTs (Ahern et al, 2020). According to this research, highly structured and commercial weight loss programmes, including WW, that help participants make real world dietary choices on a daily basis, also appear to be more effective longer term; compared with less structured or programmes where meals or supplements are provided, without recourse to enhancing knowledge and skills required to implement dietary change.

Therefore, factors likely to sustain attendance and influence adherence at WW are more likely to result in higher weight loss. The present study suggested that prior knowledge of how WW works and living in less deprived areas, both influence successful completion of the course (data not shown) and is consistent with the findings of Ahern et al (2016). Our analysis also suggests that successful weight loss (regardless of course completion) may be more likely in women with a lower starting BMI and of older age. This finding also supports the earlier observation that for the women attending the current scheme, planning for pregnancy was not a key factor influencing weight loss outcome, but general family health for existing young children might be. Further audit or research is required to understand the strength of these associations and the pathways involved.

The findings of this audit demonstrate that NHS referral to WW in Wales can meet benchmarks set for classifying effective tier 2 weight management providers. These benchmarks state that the minimum standard is “at least 30% of all participants have achieved a weight loss equal to or greater than 5% of their initial weight at the end of the intervention (using BOCF analysis)”. However, it is widely acknowledged that initial weight loss does not always lead to health benefits; moreover, researchers confirm that weight loss needs to be sustained in order to realise a beneficial health impact. Weight loss is more complex but important in the context of maternal and child health, where the potential health benefits to the unborn child are less clear. The present study is an audit of service delivery. In a real-world situation, follow up of the participants would be beneficial to determine if the weight loss was maintained and exploration of health outcomes monitored over time. Greater understanding of whether such programmes might usefully compliment existing and trusted maternity services is also recommended.

## Conclusion

Women of childbearing age or embarking on pregnancy with a healthy weight are more likely to have positive pregnancy and birthing experience, including child health outcomes. The ‘Shape Up for the Future’ NHS referral programme, delivered in Flintshire, Wales, successfully helped obese women of childbearing age (including mothers or mothers-to-be) to lose weight as part of healthy weight management. Complete attendance (10–12 sessions) is vital to support weight loss of ≥ 5% initial weight. Whilst older women with existing young children may be more motivated to complete the course, no evidence existed that intention to get pregnant in the future resulted in greater weight loss. This work also suggests that the recognised weight management intervention is potentially transferable to specific social and cultural contexts. Given the increased emphasis on maternal weight loss, weight management and obesity prevention opportunities within primary care, further research into the efficacy of commercial slimming programmes for initiating healthy weight whilst planning for pregnancy is recommended. Additionally, further audit or research is recommended before accepting that women who are planning pregnancy and taking part in commercial weight loss programmes are any less motivated.

## Appendix 1

Characteristics of WW Programme (Adapted from Jolly et al. 2010. BMC Public Health 2010, 10; 439; personal communication: Zoe Griffiths, Global Director of Nutrition, WW UK).

**Table:**

Format and content	Group sessions: 1:1 ‘orientation’ for new members – getting started and building rapport with the Coach and other new members; Wellness check in: 1:1 with Coach, discussion on monthly and weekly goals, focusing on behavioural goals and objective outcomes. Weigh in on calibrated scales; Group session following a centrally produced curriculum, covering healthier eating, being active and having a positive mind set. Facilitated by Coach; 1 Hour sessions held in community-based venues; Core programme material provided, and cross channel content to support knowledge of WW programme and motivate and inspire WW Digital tools: An integrated, multi-platform service (web/mobile app) supporting people to adopt healthier behaviours; Includes food, activity and weight tracking, recipe and meal ideas, 24/7 connection to experts and facilitated peer communities, content across programme pillars
Professional background of therapist	Trained lay-people model, fully supported by line management and a network of experts who develop and govern the programme and delivery of services. No formal qualifications required prior to training, but behavioural skills for facilitation, empathy and inspiration required and experience of building healthier habits using the WW programme Training consists of 4 meetings to observe and deliver elements, administer workshop; 3-day residential workshop; further 4 meetings delivering practical elements, and one administration workshop. In addition to a range of e-learning models. Ongoing CPD delivered across the year
Weight loss Goal	The programme is designed to deliver a weight loss of up to 1 kg per week and also supports weight maintenance
Relative Emphasis on diet and exercise	3 pillars of support, building heathier habits and healthy, sustainable weight loss: Food: Built to nudge towards a healthier pattern of eating based on national guidelines, whilst delivering freedom and flexibility for real life. Independent of weight change, the programme reduces saturated fat and sugar; Fitness: Built to inspire increased and varied physical activity based on national guidelines. Finding what is fun is key to making activity part of every-day life; Mind set: Built to deliver proven skills and techniques to help members shift their mindset because happier people make healthier choices. Rooted in science-based positive psychology, all Mindset areas have proven efficacy, practical actionable skills and strategies
Predominant behavioural change techniques used	Built on the Behaviour Change Wheel (BCW), with COM-B model at its centre. Linked to intervention functions and operationalised through the Behaviour Change Techniques (using 63/93 techniques in Michie’s taxonomy) offering a comprehensive and systematic behaviour change approach Participants are given a ‘budget’ to support their food choices and personal goals for fitness

## Abbreviations

BMI: Body Mass Index
BOCF: Baseline observation carried forward
Chi^2^: Chi-square
CI: Confidence interval
IQR: Interquartile range
ITT: Intention to treat
LOCF: Last observation carried forward
P: Probability
R^2^: Regression coefficient
WW: Weight Watchers

## Footnotes

1 Lower Super Output Area (LSOA) is the geographic unit used in the Welsh Index of Multiple Deprivation (WIMD). LSOAs are built from groups of Output Areas (OAs) used for the 2001 Census http://gov.wales/docs/statistics/lsoamaps/lsoa.htm

2 WIMD is the official measure of relative deprivation for small areas in Wales http://gov.wales/splash?orig=/statistics-and-research/welsh-index-multiple-deprivation

## References

[CR1] Ahern AL, Olson AD, Aston LM, Jebb SA (2011). WW on prescription: An observational study of weight change among adults referred to WW by the NHS. BMC Public Health.

[CR2] Ahern AL, Aveyard P, Boyland EJ, Halford JC, Jebb SA (2016). Inequalities in the uptake of weight management interventions in a pragmatic trial: an observational study in primary care. British Journal of General Practice.

[CR3] Ahern AL, Wheeler GM, Aveyard P, Boyland EJ, Halford JCG, Mander AP, Woolston J, Thomson AM, Tsiountsioura M, Cole D, Mead BR, Irvine L, Turner D, Suhrcke M, Pimpin L, Retat L, Jaccard A, Webber L, Cohn SR, Jebb SA (2017). Extended and standard duration weight-loss programme referrals for adults in primary care (WRAP): A randomised controlled trial. Lancet.

[CR4] Ahern AL, Griffin SJ, Wheeler GM, Sharp SJ, Aveyard P, Boyland EJ, Halford JC, Jebb SA (2020). The effect of referral to an open-group behavioural weight-management programme on the relative risk of normoglycaemia, non-diabetic hyperglycaemia and type 2 diabetes: Secondary analysis of the WRAP trial. Diabetes, Obesity & Metabolism.

[CR5] Basu A, Kennedy L, Tocque K (2014). Eating for 1, Healthy and Active for 2: Feasibility of delivering novel compact training for midwives to build knowledge and confidence in giving nutrition advice during pregnancy. BMC Pregnancy and Childbirth..

[CR6] Boudet-Berquier J, Salanave B, Desenclos J, Castetbon K (2017). Sociodemographic factors and pregnancy outcomes associated with pre-pregnancy obesity: Effect modification of parity in the nationwide Epifane birth-cohort. BMC Pregnancy and Childbirth.

[CR7] Centre for Maternal and Child Enquiries (CMACE) (2010). Maternal obesity in the UK: Findings from a national project.

[CR8] Centre for Maternity and Child Enquiries (CMACE). (2011). *Perinatal Mortality 2009*. CMACE. ISBN: 978-0-9558055-7-8

[CR9] Chen C, Xu X, Yan Y (2018). Estimated global overweight and obesity burden in pregnant women based on panel data model. PLoS ONE.

[CR11] Confidential Enquiry into Maternal and Child Health (CEMACH). (2004): Why Mothers Die 2000–2002. *In: The Sixth Report of the Confidential Enquiries into Maternal Deaths in the United Kingdom.* London: Royal College of Obstetricians and Gynaecologists. 2004. https://stratog.rcog.org.uk/sites/default/files/Gynaecological%20emergencies/CEMACH_Why_Mothers_Die_00-02_2004.pdf. Accessed 21 July 2021

[CR10] Confidential Enquiry into Maternal and Child Health (CEMACH). (2007): *Saving mothers’ lives: reviewing maternal deaths to make motherhood safer- 2003–2005. The Seventh Report on Confidential Enquiries into Maternal Deaths in the United Kingdom*. London: CEMACH. ISBN: 978-0-9533536-9-9

[CR12] Denison, F. C., Aedla, N. R., Keag, O., Hor, K., Reynolds, R. M., Milne, A., Diamond, A., & on behalf of the Royal College of Obstetricians and Gynaecologists. (2018). *Care of women with obesity in pregnancy*. Green-top Guideline No. 72. BJOG 2018. 10.1111/1471-0528.15386. 10.1111/1471-0528.15386 30465332

[CR13] Department of Health. (2013). *Developing a specification for lifestyle weight management services*. Retrieved July 21, 2021, from www.gov.uk/government/publications/best-practice-guidance-for-weight-management-services.

[CR14] Furber CM, McGowan L (2011). A qualitative study of the experiences of women who are obese and pregnant in the UK. Midwifery..

[CR15] Gaillard R (2015). Maternal obesity during pregnancy and cardiovascular development and disease in the offspring. European Journal of Epidemiology..

[CR16] Godfrey KM, Reynolds RM, Prescott SL, Nyirenda M, Jaddoe VW, Eriksson JG, Broekman BF (2017). Influence of maternal obesity on the long-term health of offspring. The Lancet. Diabetes & Endocrinology.

[CR17] Gudzune KA, Doshi RS, Mehta AK, Chaudrey ZW, Jacobs DK, Vakil RM, Lee CJ, Bleich SN, Clark JM (2015). Efficacy of commercial weight loss programs: An updated systematic review. Annals of Internal Medicine.

[CR18] Health and Social Care Information Centre. (2018). *Health Survey for England 2017: Adult, Child overweight, and obesity*. Surrey. Health and Social Care Information Centre.

[CR19] Heshka S, Anderson JW, Atkinson RL, Greenway FL, Hill JO, Phinney SD, Kolotkin RL, Miller Krovach K, Pi-Sunyer FX (2003). Weight loss with self-help compared with a structured commercial program: A randomized trial. JAMA.

[CR20] Hilden K, Hanson U, Persson M, Fadl H (2016). Overweight and obesity: A remaining problem in women treated for severe gestational diabetes. Diabetic Medicine.

[CR21] Jolley K, Daley A, Adab P, Lewis A, Denley J, Beach J, Aveyard P (2010). A randomised controlled trial to compare a range of commercial or primary care led weight reduction programmes with a minimal intervention control for weight loss in obesity: The Lighten Up trial. BMC Public Health.

[CR22] Kocełak P, Chudek J, Naworska B, Sosnowska M, Kotlarz B, Mazurek M, Madej P, Skrzypulec-Plinta V, Skałba P, Glinianowicz M (2012). Psychological disturbances and quality of life in obese and infertile women and men. International Journal of Endocrinology.

[CR23] Marmot (2010). Fair society, healthy lives.

[CR24] Meehan S, Beck CR, Mair-Jenkins J, Leonardi-Bee J, Puleston R (2014). Maternal obesity and infant mortality: A meta-analysis. Pediatrics.

[CR25] Morgan KL, Rahman MA, Macey S (2014). Obesity in pregnancy: A retrospective prevalence-based study on health service utilisation and costs on the NHS. BMJ Open.

[CR26] Moyer VA (2012). Screening for and management of obesity in adults: U.S. Preventive Services Task Force Recommendation Statement. Annals of Internal Medicine.

[CR27] NHS Welsh Risk Pool Services. (2012). *Clinical Area 1, Maternity 2011–12.* Retrieved July 21, 2021, from http://www.nwssp.wales.nhs.uk/archive.

[CR28] NICE. (2014b). Nice Public Health Guidance 27. Weight management before, during and after pregnancy. 2010. NICE. Retrieved July 21, 2021, from https://www.nice.org.uk/guidance/ph27.

[CR29] NICE. (2018). *Public Health Guidance Maternal and Child Nutrition* (PH11) March 2018 (updated 2014). NICE.

[CR30] NICE. (2014a). *Obesity: identification, assessment an management. Clinical guideline [CG 189]* NICE. London. November 2014. Retrieved July 21, 2021, from https://www.nice.org.uk/guidance/cg189/chapter/1-Recommendations#behavioural-interventions.

[CR31] Nyman VMK, Prebensen AK, Flensner GEM (2010). Obese women’s experiences of encounters with midwives and physicians during pregnancy and childbirth. Midwifery.

[CR32] Powell J, Hughes C (2012). Antenatal interventions that support obese pregnant women. British Journal of Midwifery.

[CR33] Reynolds AJ (1998). Confirmatory program evaluation: A method for strengthening causal inference. American Journal of Evaluation..

[CR34] Smith D, Cooke A, Lavender T (2012). Maternal obesity is the new challenge; a qualitative study of health professionals’ views towards suitable care for pregnant women with a Body Mass Index (BMI) ≥30 kg/m2. Pregnancy and Childbirth..

[CR35] The Centre for Maternal and Child Enquiries. (2011). *Saving Mothers Lives: Reviewing maternal deaths to make motherhood safer* (2006–2008). The Centre for Maternal and Child Enquiries. 10.1093/bja/aer192 21757549

[CR36] The Royal College of Midwives. (2018). *Call for clear guidance on healthy weight management in pregnancy from the Royal College of Midwives and Nice Public Health Guidance Maternal and Child Nutrition*. https://www.rcm.org.uk/news-views-and-analysis/news/call-for-clear-guidance-on-healthy-weight-management-in-pregnancy-from

[CR37] Wee CC, Hamel MB, Davis RB (2004). Assessing the value of weight loss among primary care patients. Journal of General Internal Medicine.

[CR38] Wojcicki JM (2011). Maternal Pre pregnancy body mass index and initiation of breastfeeding: A review of the literature. Journal of Women’s Health..

